# Efficacy and safety of VEGF/VEGFR inhibitors for platinum-resistant ovarian cancer: a systematic review and meta-analysis of randomized controlled trials

**DOI:** 10.1186/s12905-023-02879-y

**Published:** 2024-01-13

**Authors:** Danxue Huang, Liyuan Ke, Hongxia Cui, Su Li, Feilong Sun

**Affiliations:** 1 Department of Pharmacy, Cancer Hospital of China Medical University, Liaoning Cancer Hospital & Institute, Shenyang, China; 2 Jiangsu Hengrui Pharmaceuticals Co., LTD, Lianyungang, China

**Keywords:** VEGF/VEGFR inhibitors, Platinum-resistant, Ovarian cancer, meta-analysis, Randomized controlled trials

## Abstract

**Background:**

Almost all patients with ovarian cancer will experience relapse and eventually develop platinum-resistant. The poor prognosis and limited treatment options have prompted the search for novel approaches in managing platinum-resistant ovarian cancer (PROC). Therefore, a meta-analysis was conducted to evaluate the efficacy and safety of combination therapy with vascular endothelial growth factor (VEGF) /VEGF receptor (VEGFR) inhibitors for PROC.

**Methods:**

A comprehensive search of online databases was conducted to identify randomized clinical trials published until December 31, 2022. Pooled hazard ratios (HR) was calculated for overall survival (OS) and progression-free survival (PFS), while pooled odds ratio (OR) was calculated for objective response rate (ORR) and treatment-related adverse events (TRAEs). Subgroup analysis was further performed to investigate the source of heterogeneity.

**Results:**

In total, 1097 patients from eight randomized clinical trials were included in this meta-analysis. The pooled HRs of OS (HR = 0.72; 95% CI: 0.62–0.84, *p* < 0.0001) and PFS (HR = 0.52; 95% CI: 0.45–0.59, *p* < 0.0001) demonstrated a significant prolongation in the combination group compared to chemotherapy alone for PROC. In addition, combination therapy demonstrated a superior ORR compared to monotherapy (OR = 2.34; 95%CI: 1.27–4.32, *p* < 0.0001). Subgroup analysis indicated that the combination treatment of VEGF/VEGFR inhibitors and chemotherapy was significantly more effective than monochemotherapy in terms of OS (HR = 0.71; 95% CI: 0.61–0.84, *p* < 0.0001), PFS (HR = 0.49; 95% CI: 0.42–0.57, *p* < 0.0001), and ORR (OR = 2.97; 95% CI: 1.89–4.67, *p* < 0.0001). Although the combination therapy was associated with higher incidences of hypertension, mucositis, proteinuria, diarrhea, and hand-foot syndrome compared to monochemotherapy, these toxicities were manageable and well-tolerated.

**Conclusions:**

The meta-analysis demonstrated that combination therapy with VEGF/VEGFR inhibitors yielded better clinical outcomes for patients with PROC compared to monochemotherapy, especially when combined with chemotherapy. This analysis provides more treatment options for patients with PROC.

**Systematic review registration:**

[https://www.crd.york.ac.uk/PROSPERO], Prospective Register of Systematic Reviews (PROSPERO), identifier: CRD42023402050.

**Supplementary Information:**

The online version contains supplementary material available at 10.1186/s12905-023-02879-y.

## Introduction

Ovarian cancer (OC) is the seventh most common cancer in women and the eighth leading cause of cancer-related death worldwide [1]. At the time of initial diagnosis, over 70% of patients present with advanced disease due to the presence of atypical early symptoms [2]. Currently, for patients with a new diagnosis, the standard first-liner treatment involves cytoreductive surgery combined with platinum-based systematic chemotherapy, with or without the addition of bevacizumab. However, at first relapse, approximately 25% of patients develop platinum-resistant ovarian cancer (PROC), and nearly all patients will experience relapse and eventually develop platinum-resistant [3]. PROC is associated with a poor prognosis and an overall survival (OS) of less than 12 months, presenting a significant therapeutic challenge [4]. In the platinum-resistant setting, monotherapy with docetaxel, paclitaxel, topotecan or pegylated liposomal doxorubicin (PLD) remains the primary therapeutic option, but it results in a remarkably short survival, highlighting the urgent need for better treatment options. Furthermore, several trials have demonstrated that combining chemotherapy agents leads to increased adverse events without improving clinical benefit for PROC [5–7].

Tumor angiogenesis has been established as a hallmark of tumor development, growth, and metastasis. This complex process involves multiple signaling pathways. Vascular endothelial growth factor (VEGF), an important driver of angiogenesis in solid tumors, binds to VEGF receptor-1 or -2 (VEGFR-1/VEGFR-2) on target cells [8], thereby activating intracellular tyrosine kinase signaling. VEGF promotes the recruitment of circulating endothelial progenitor cells from the bone marrow and facilitates endothelial cell survival, differentiation, and proliferation during angiogenesis. Angiogenesis also plays a crucial role in the pathogenesis of OC by promoting tumor proliferation and metastasis [9, 10]. The presence of extensive neovascularization is closely associated with a poor prognosis in OC. Anti-VEGF therapy has emerged as a promising therapeutic target with potential clinical benefits for patients with OC, including those with platinum-resistant disease [11–14]. Recently, various anti-VEGF therapies, such as anti-VEGF monoclonal antibodies (e.g., bevacizumab) and VEGF-R tyrosine kinase inhibitors (e.g., sorafenib, pazopanib, apatinib, cediranib, anlotinib), have been evaluated in OC patients [15].

The AURELIA trial, a randomized phase III trial, demonstrated a significant improvement in progression-free survival (PFS) in PROC patients when treated with a combination of bevacizumab and chemotherapy compared to monochemotherapy (hazard ratio (HR) = 0.48; 95% CI: 0.38–0.60). The median PFS was 6.7 months with the combined regimen versus 3.4 months with monochemotherapy. The objective response rate (ORR) also increased by 15.5% compared to chemotherapy alone. However, there was no statistically significant improvement in OS when bevacizumab was combined with chemotherapy (HR = 0.85; 95% CI: 0.66–1.08, *p* < 0.17) [16]. Bevacizumab has been approved by the Food and Drug Administration (FDA) for PROC. Other anti-VEGF agents, such as apatinib, have also shown preliminary evidence of efficacy when combined with chemotherapy for PROC. Wang et al. reported that treatment with apatinib plus PLD resulted in a clinically meaningful improvement in PFS (HR = 0.44; 95% CI: 0.28–0.71, *p* < 0.001). The median PFS was 5.8 months for apatinib plus PLD versus 3.3 months for PLD alone. The median OS was 23.0 months versus 14.4 months for apatinib plus PLD and PLD alone, respectively (HR = 0.66; 95% CI: 0.40–1.09) [17].

Previous meta-analyses have demonstrated that combination therapy offers improved survival benefits compared to chemotherapy alone in ovarian cancer patients [18–21]. However, there is a lack of specific meta-analysis focusing on platinum-resistant patients. Given the clinical uncertainty and inconsistent efficacy related to VEGF/VEGFR inhibitors in PROC, a systematic review and meta-analysis was conducted to overcome the limitations of individual studies and provide a more accurate estimation of the efficacy and safety of VEGF/VEGFR inhibitors in PROC.

## Materials and methods

This systematic review and meta-analysis adhered to the Preferred Reporting Items for Systematic Reviews and Meta-Analyses (PRISMA) guidelines. The study was registered with the International Prospective Register of Systematic Reviews (PROSPERO CRD42023402050).

### Data source and search strategy

Eligible studies were identified by searching databases including Cochrane Library, PubMed, Embase, and Web of Science. The search covered the period from inception to December 2022. The main search terms associated with therapy included (anti-angiogenic OR targeted therapy OR molecular targeted therapy OR bevacizumab OR nintedanib OR pazopanib OR cediranib OR sorafenib OR apatinib OR anlotinib OR lenvatinib OR ramolumab OR VEGF OR VEGFR OR vascular endothelial growth factor). The terms related to the disease included ovarian cancer OR ovarian neoplasm. Subsequently, the reference lists of all relevant articles were also browsed.

### Study selection

The following criteria were used to screen potential trials: (1) prospective phase II and phase III randomized controlled trials (RCTs); (2) patients with OC, peritoneal cancer (PC), or fallopian tube cancers (FTC) that had progressed during platinum therapy (platinum-refractory) or within 6 months of platinum-containing therapy (platinum-resistant); (3) comparison with therapy combining VEGF/VEGFR inhibitors with other drugs (chemotherapy or Poly (ADP-ribose) polymerase (PARP) inhibitors) and chemotherapy alone; (4) the study’s clinical outcomes included at least one of OS, PFS, ORR, and treatment-related adverse events (TRAEs); (5) Only studies published in English were included. The following criteria were excluded: reviews, fundamental studies, editorials, animal studies, comments, and case reports.

### Data extraction and quality assessment

For each eligible study, we extracted the following information: (1) general study information (study name, publication year, first author, study design, trial phase, sample size); (2) basic patient information (region, age, Eastern Cooperative Oncology Group (ECOG) performance status, primary tumor site); (3) control and intervention group. The main outcomes assessed were OS, PFS, ORR, and TRAEs. The risk of bias and methodological quality assessment was performed using the Cochrane Collaboration’s tool in RevMan5.4.

### Statistical analysis

Statistical analysis was conducted using Stata 14.0 and RevMan5.4. Pooled odds ratios (ORs) and 95% confidence interval CI were calculated for ORR and TRAEs, while pooled HRs and 95% (CI) were calculated for OS and PFS. With *I*^*2*^ > 50% and *p* < 0.05 indicating statistically significant heterogeneity [22], a random-effects model was utilized to calculate the HR and OR; otherwise, the fixed-effects model was employed.

Publication bias assessment, sensitivity analysis, and subgroup analysis were conducted to further explore the source of heterogeneity. Begg’s test was performed to evaluate publication bias, and the results indicated the absence of publication bias with *p* > 0.05 [23]. The symmetry of the funnel plot was also visually observed to assess publication bias. Additionally, a sensitivity analysis was carried out by excluding each study to observe any changes in the pooled HR and OR. Subgroup analysis took into account factors such as region, combination therapeutic agents, trial phase, ECOG performance status, publication year, and primary tumor site.

## Results

### Study selection and characteristics

**Fig. 1:**
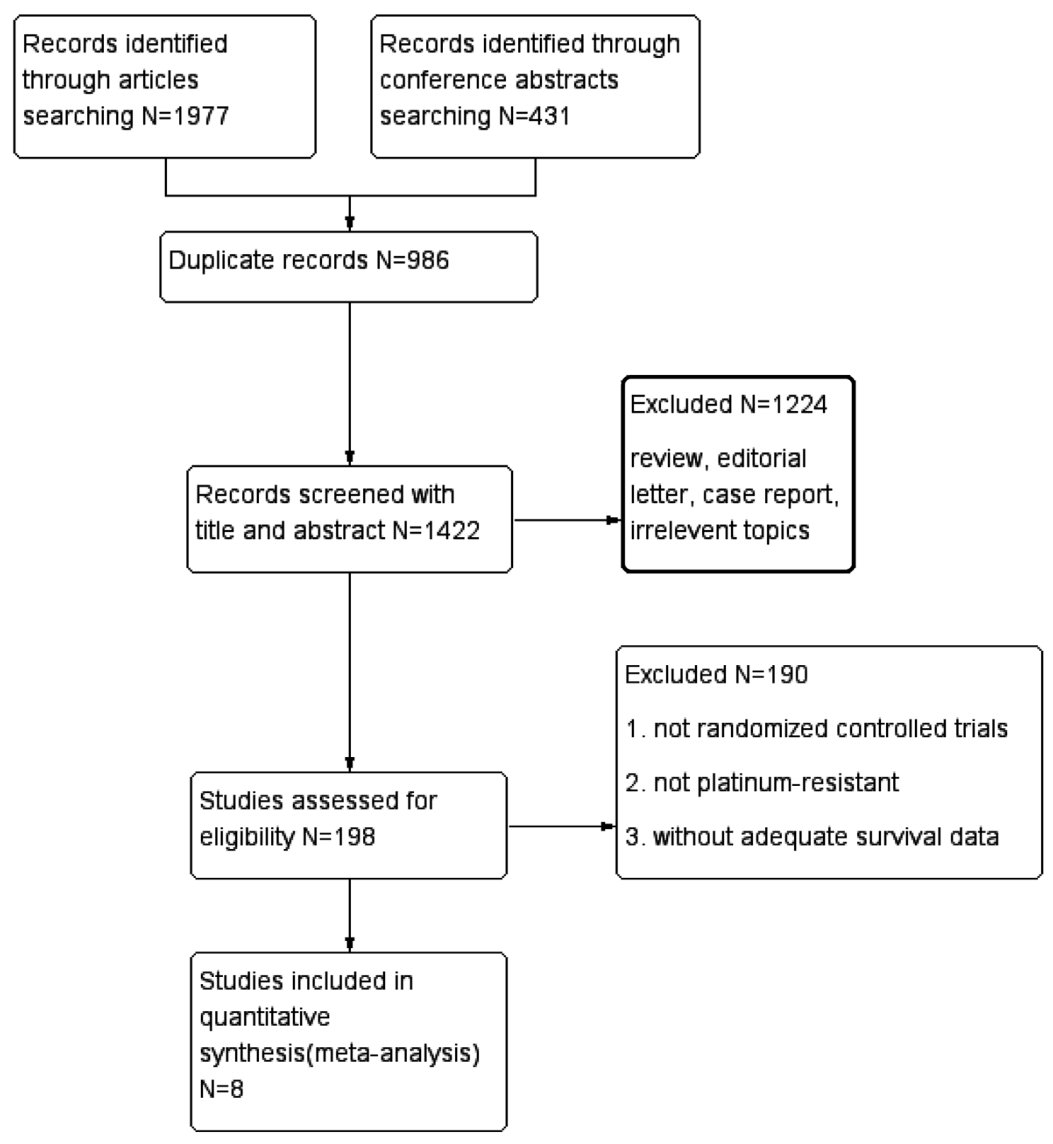
Flow diagram of the screening and selection process

A total of 2408 potentially relevant trials were collected through independent evaluation by two authors. After removing irrelevant and duplicate studies, the initial search yielded 1422 abstracts and articles. Finally, eight studies were included (Fig. 1) [16, 17, 24–29].

Table 1 recorded the general information of the studies, therapeutic regimens, and baseline characteristics of the patients. Seven studies were prospective phase II RCTs, and one was a prospective phase III RCT. The studies were published between 2014 and 2022, and a total of 1097 patients were available for the meta-analysis, with a mean age of approximately 61 years.

**Table 1:** Main characteristic of the eligible studies in the meta-analysis

Author	Year	Study Phase/design	Numbers of parents	Median age	Region	Arm	Median OS	HR (95%CI)	Median PFS	HR (95%CI)	ORR	Primary tumor site
Chekerov et al.	2018	II/RCT	85/89	59/58	Germany	Sorafenib + topotecan vs. placebo + topotecan	17.1 vs. 10.1	0.65 ( 0.45–0.93)	6.7 vs. 4.4	0.60 (0.43–0.83)	30.8% vs. 12%	OC FTC PC
Colombo et al.	2022	II/RCT	41/41	64/63	Italy	Cediranib + olaparib vs. paclitaxel	11.6 vs. 9.3	0.86 ( 0.5–1.46)	5.6 vs. 3.1	0.76 (0.50–1.14)	15.4% vs. 37.5%	OC FTC PC
Pignata et al.	2014	II/RCT	37/37	56/58	Italy	paclitaxel + pazopanib vs. paclitaxel	19.1 vs. 13.7	0.60 (0.32–1.13)	6.4 vs. 3.5	0.42 (0.25–0.69)	55.6% vs. 25%	OC FTC PC
Pujade-Lauraine et al.	2014	III/RCT	179/182	62/61	European	chemotherapy + bevacizumab vs. chemotherapy	16.6 vs. 13.3	0.85 (0.66–1.08)	6.7 vs. 3.4	0.48 (0.38–0.60)	30.9% vs. 12.6%	OC FTC PC
Roque et al.	2021	II/RCT	39/37	67/67	United States	ixabepilone + bevacizumab vs. ixabepilone	10.0 vs. 6.0	0.52 (0.31–0.87)	5.5 vs. 2.2	0.33 (0.19–0.55)	30.8% vs. 8.1%	OC FTC PC
Sharma et al.	2021	II/RCT	37/38	54/53	India	Pazopanib + etoposide + cyclophosphamide vs. etoposide + cyclophosphamide	- vs. 11.2	0.64 (0.25–1.65)	5.1 vs. 3.4	0.67 (0.34–1.30)	54.1% vs. 55.3%	OC
Shoji et al.	2021	II/RCT	52/51	60/61	Japan	chemotherapy + bevacizumab vs. chemotherapy	15.3 vs. 11.3	0.67 (0.38–1.17)	4.0 vs. 3.1	0.54 (0.32–0.90)	25% vs. 13.7%	OC
Wang et al.	2022	II/RCT	78/74	54/56	China	Apatinib + PLD vs. PLD	23.0 vs. 14.4	0.66 (0.40–1.09)	5.8 vs. 3.3	0.44 (0.28–0.71)	43.1% vs. 10.9%	OC FTC PC

### Risk of bias

Seven studies were deemed to have a high risk of bias in blinding participants and personnel, while five studies had an unclear risk of bias in blinding outcome assessment, and one study had a high risk. The remaining studies were rated as having a low risk of bias (Figure S1).

### Meta-analysis of OS and PFS

**Fig. 2:**
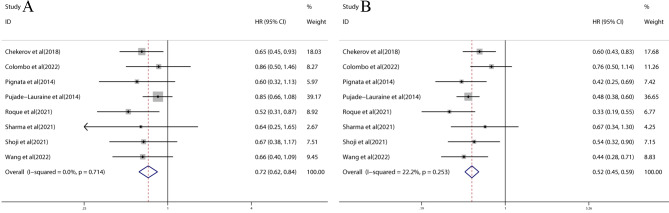
Forest plots of OS **(A)** and PFS **(B)** of combination therapy with VEGF/VEGFR inhibitors

The pooled effects of HR for OS and PFS were available for all eight trials. The results demonstrated that combination therapy with VEGF/VEGFR inhibitors had a significantly better OS than chemotherapy (HR = 0.72; 95% CI: 0.62–0.84, *p* < 0.0001) (Fig. 2A). Compared to chemotherapy, combination therapy with VEGF/VEGFR inhibitors resulted in a significant improvement in PFS (HR = 0.52; 95% CI: 0.45–0.59, *p* < 0.0001) (Fig. 2B). Additionally, there were no significant heterogeneities observed in OS and PFS results among the included studies (*I*^*2*^ = 0% and 22.2%, respectively).

### Meta-analysis of ORR

**Fig. 3:**
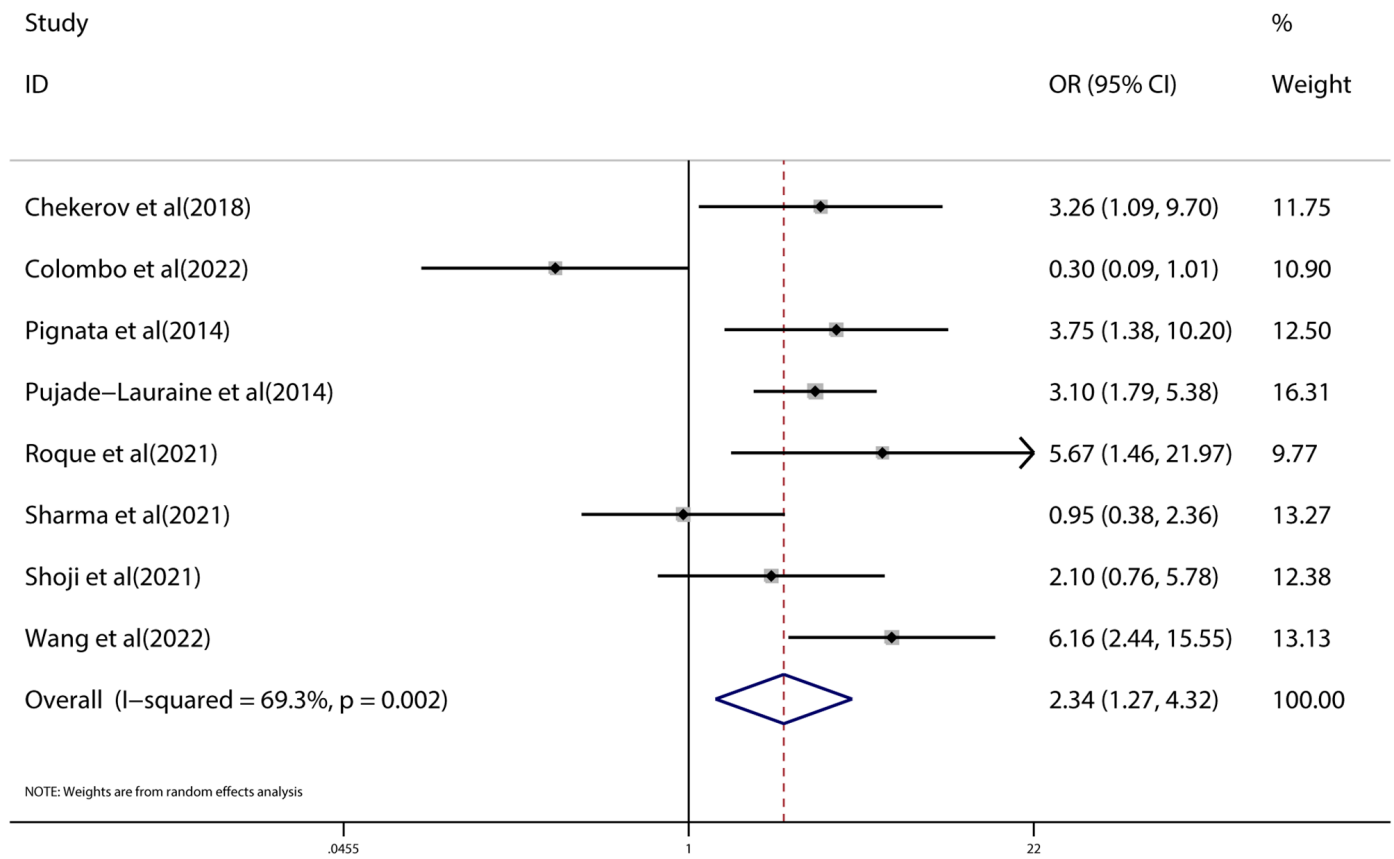
Forest plot of ORR of combination therapy with VEGF/VEGFR inhibitors

All eight trials with PROC reported ORR. Interestingly, the group of combination therapy exhibited respectable ORRs compared to chemotherapy (OR = 2.34; 95%CI: 1.27–4.32, *p* < 0.0001). There was a high degree of heterogeneity among different studies for ORR (*I*^*2*^ = 69.3%, *p* = 0.002). Subgroup analyses were conducted to determine the source of heterogeneity. A pooled analysis of ORR in patients with PROC was presented in Fig. 3.

### Subgroup analysis for OS

Subgroup analyses were conducted based on stratification factors including region, combination therapeutic agents, trial phase, ECOG performance status, publication year, and primary tumor site. The results were displayed in Table 2 and Figure S2. In the subgroup of combination therapeutic agents, a better OS benefit was revealed in combination treatment with chemotherapy (HR = 0.71; 95% CI: 0.61–0.84, *p* < 0.0001). Patients with an ECOG performance status of 0 to 2 showed greater OS benefit in the combination treatment group compared to monochemotherapy (HR = 0.72; 95% CI: 0.61–0.85, *p* < 0.0001). Furthermore, no significant heterogeneity was observed in any of the subgroups.

**Table 2:** The subgroup analysis for OS in patients with PROC

Subgroup		Pooled OS		Heterogeneity	
		HR[95% CI]	*p*	*I* ^*2*^	*p*
Combination therapeutic agents	Chemotherapy	0.71[0.61, 0.84]	0.000	0%	0.660
	PARP inhibitors	0.86[0.50, 1.47]	0.581	-	-
Trial phase	phase II	0.65[0.54, 0.79]	0.000	0%	0.933
	phase III	0.85[0.66, 1.09]	0.196	-	-
Region	non-Asia	0.74[0.62, 0.88]	0.001	5.4%	0.376
	Asia	0.66[0.47, 0.94]	0.02	0%	0.997
ECOG	0–2	0.72[0.61, 0.85]	0.000	0%	0.539
	0–4	0.76[0.52, 1.13]	0.173	0%	0.529
Primary tumor site	OC, FTC, PC	0.73[0.62, 0.86]	0.000	0%	0.492
	OC	0.66[0.41,1.07]	0.094	0%	0.935
Publication year	within 5 years	0.66[0.53, 0.81]	0.000	0%	0.880
	5 years ago	0.81[0.65, 1.02]	0.075	1.6%	0.313

### Subgroup analysis for PFS

The subgroups of region, trial phase, ECOG performance status, publication year, and primary tumor site suggested that combination therapy exhibited better PFS than those receiving chemotherapy alone (Table 3 and Figure S3). Compared to the chemotherapy group, only the subgroup of combination treatment with PARP inhibitors exhibited no significant difference (HR = 0.76, 95% CI: 0.50–1.15, *p* = 0.192). The heterogeneity within each subgroup was no significant (*p* > 0.05).

### Subgroup analysis for ORR

The results were presented in Table 4 and Figure S4. In the subgroup analysis of combination therapeutic agents, the combination therapy with chemotherapy showed a greater benefit in terms of ORR (OR = 2.97; 95% CI: 1.89–4.67, *p* < 0.0001). In the subgroup analysis of ECOG performance status, significant benefit of ORR was observed in patients with ECOG scores of 0 to 2 (OR = 3.14; 95% CI: 1.87–5.27, *p* < 0.0001). The heterogeneities of the two subgroups were reduced.

### Meta-analysis of TRAEs

**Fig. 4:**
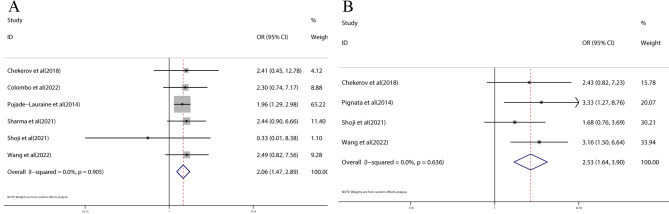
Forest plots of any grade TRAEs **(A)** and grade 3–4 TRAEs **(B)**

Six trials reported the incidences of any grade TRAEs and four trials reported grade 3–4 TRAEs. For both any grade TRAEs (OR = 2.06; 95% CI: 1.47–2.89, *p* < 0.0001) and grade 3–4 TRAEs (OR = 2.53; 95% CI: 1.64–3.90, *p* < 0.0001), the combination therapy with VEGF/VEGFR inhibitors was associated with significantly higher incidences compared to chemotherapy (Fig. 4).

The meta-analysis indicated that compared to chemotherapy, combination therapy had a higher incidence of any grade hypertension (OR = 4.38, 95%CI 1.28–14.93, *p* = 0.018), mucositis (OR = 3.20, 95%CI 1.25–8.16, *p* = 0.015), proteinuria (OR = 6.15, 95%CI 1.75–21.59, *p* = 0.005), diarrhea (OR = 3.14, 95%CI 1.36–7.25, *p* = 0.007), and hand-foot syndrome (OR = 6.52, 95%CI 1.02–41.70, *p* = 0.048). There was no statistical difference in the incidence of fatigue (OR = 1.64, 95%CI 0.87–3.10, *p* = 0.124), nausea (OR = 1.36, 95%CI 0.72–2.54, *p* = 0.341), and vomiting (OR = 1.74, 95%CI 0.76–4.02, *p* = 0.192) (Table 5 and Figure S5).

**Table 3:** The subgroup analysis for PFS in patients with PROC

Subgroup		Pooled OS		Heterogeneity	
		HR[95% CI]	*p*	*I* ^*2*^	*p*
Combination therapeutic agents	Chemotherapy	0.49[0.42,0.57]	0.000	0%	0.525
	PARP inhibitors	0.76[0.50,1.15]	0.192	-	-
Trial phase	phase II	0.54[0.45,0.64]	0.000	28.7%	0.209
	phase III	0.48[0.38,0.60]	0.000	-	-
Region	non-Asia	0.51[0.44,0.60]	0.000	49.6%	0.094
	Asia	0.52[0.38,0.70]	0.000	0%	0.588
ECOG	0–2	0.49[0.42,0.57]	0.000	0%	0.416
	0–4	0.67[0.48,0.92]	0.013	2.6%	0.311
Primary tumor site	OC, FTC, PC	0.51[0.44,0.59]	0.000	40%	0.139
	OC	0.59[0.39,0.88]	0.01	0%	0.618
Publication year	within 5 years	0.55[0.46,0.67]	0.000	32.4%	0.193
	5 years ago	0.47[0.38,0.58]	0.000	0%	0.638

**Table 4:** The subgroup analysis for ORR in patients with PROC

Subgroup		Pooled OS		Heterogeneity	
		OR[95% CI]	*p*	*I* ^*2*^	*p*
Combination therapeutic agents	Chemotherapy	2.97[1.89,4.67]	0.000	39.9%	0.125
	PARP inhibitors	0.30[0.09,1.01]	0.051	-	-
Trial phase	phase II	2.22[1.03, 4.75]	0.041	72.7%	0.001
	phase III	3.10[1.79,5.38]	0.000	-	-
Region	non-Asia	2.36[1.01,5.49]	0.047	72.8%	0.005
	Asia	2.31[0.77,6.91]	0.136	75%	0.018
ECOG	0–2	3.14[1.87,5.27]	0.000	47.3%	0.091
	0–4	0.82[0.12,5.45]	0.836	82.8%	0.016
Primary tumor site	OC, FTC, PC	2.80[1.34,5.84]	0.006	71.3%	0.004
	OC	1.37[0.63,2.95]	0.427	22.3%	0.257
Publication year	within 5 years	2.02[0.83,4.91]	0.119	75.9	0.001
	5 years ago	3.24[2.00,5.25]	0.000	0%	0.745

**Table 5:** The TRAEs of combination therapy with VEGF/VEGFR inhibitors in PROC

TRAEs (any grade)	Pooled ES		Heterogeneity	
	OR[95% CI]	***p***	I^2^	***p***
Hypertension	4.38[1.28–14.93]	0.018	72.1%	0.003
Mucositis	3.20[1.25–8.16]	0.015	33.0%	0.225
Proteinuria	6.15[1.75–21.59]	0.005	0.0%	0.824
Diarrhea	3.14[1.36–7.25]	0.007	42.9%	0.154
Hand-foot syndrome	6.52[1.02–41.70]	0.048	76.6%	0.014
Fatigue	1.64[0.87–3.10]	0.124	61.6%	0.034
Nausea	1.36[0.72–2.54]	0.341	59.1%	0.044
Vomiting	1.74[0.76–4.02]	0.192	55.0%	0.064

### Sensitivity analysis and publication bias

Microvariation was observed in the sensitivity analysis when each trial was removed in turn (Figure S6). There was no publication biases according to Begg’s test (OS, *p* = 0.107; PFS, *p* = 0.998; ORR, *p* = 0.617), and the funnel plots were mostly symmetric (Figure S7).

## Discussion

OC is often asymptomatic until it reaches an advanced stage, resulting in delayed diagnosis and poor prognosis. The current screening programs for OC diagnosis are inadequate [31]. PROC remains a significant challenge for clinical diagnosis and treatment due to the extreme cellular heterogeneity and the expression of various resistance and immune evasion mechanisms in this advanced stage of tumor complexity [25]. Combination therapy with VEGF/VEGFR inhibitors has shown a higher likelihood of being the most effective treatment compared to chemotherapy. Recent studies have reported encouraging results, particularly in terms of PFS, for several combination strategies involving VEGF/VEGFR inhibitors in PROC. However, the OS outcomes have been uncertain and inconsistent [16, 17]. To address this, a meta-analysis was conducted, which included eight randomized controlled trials in PROC, and demonstrated better OS, PFS, and ORR outcomes with VEGF/VEGFR inhibitors compared to monochemotherapy. Furthermore, heterogeneity was observed in terms of ORR among the included studies.

Subgroup analyses were performed for OS, PFS, and ORR, considering various stratification factors such as region, combination therapeutic agents, trial phase, ECOG performance status, publication year, and primary tumor site. Regardless of OS, PFS, or ORR, combination therapy with chemotherapy showed greater benefits in the subgroup analysis of combination therapeutic agents. Only one trial included combined PARP inhibitors therapy (cediranib plus olaparib), but it failed to demonstrate any superiority in efficacy compared to the standard treatment for patients with PROC [25]. Some studies have reported that cediranib induces the down-regulation of certain genes in the homologous recombination system, which synergistically enhances the effect of olaparib [32, 33]. Liu et al. demonstrated that the combination of cediranib and olaparib significantly prolonged PFS compared to olaparib alone in platinum-sensitive OC patients (HR = 0.50). Additionally, in the gBRCA/unknown-subset, the combination therapy showed significantly improved OS compared to olaparib alone (37.8 versus 23.0 months, *p* = 0.047) [34]. However, disappointing results were observed for both OS and PFS in the platinum resistance trials included in our analysis [25]. It should be noted that due to the limited number of trials, the accuracy of subgroup analysis may be insufficient. It is necessary to explore randomized controlled trials of new combinations of PARP inhibitors with various drugs, such as anti-angiogenesis agents, immune checkpoint inhibitors, or other inhibitors of DNA damage response pathways [35].

The analysis of TRAEs revealed that the combination therapy had significantly higher incidences of both any grade TRAEs and grade 3–4 TRAEs compared to monochemotherapy. These findings were consistent with the previously published safety profile of VEGF/VEGFR inhibitors in OC and other solid tumors [36–41], and no new safety concerns were identified. Most of the TRAEs reported were of grade 1–2, indicating that the adverse events were manageable. Only four trials reported the incidence of grade 3–4 TRAEs. Among them, paclitaxel plus pazopanib treatment had a higher incidence (OR = 3.33, 95% CI: 1.27–8.76), while bevacizumab plus chemotherapy had a lower incidence (OR = 1.68, 95% CI: 0.76–3.69). Combination therapy was associated with a higher incidence of any grade hypertension, mucositis, proteinuria, diarrhea, and hand-foot syndrome. Hypertension is a common adverse effect of VEGF inhibitors, with an incidence of approximately 30% in various clinical trials, and moderate hypertension occurring in 3–16% of cases. Mucositis is another common adverse effect of anti-VEGF therapy, characterized by symptoms such as pain, difficulty swallowing and pronunciation. Mucositis typically manifests 7–10 days after the initiation of treatment, and in the absence of concurrent bacterial, viral, or fungal infections, it is self-limiting and resolves spontaneously within 2–4 weeks. The mechanism underlying proteinuria production involves the regulation of glomerular vascular permeability by the VEGF signaling pathway. Inhibition of VEGF can result in the destruction of glomerular endothelial cells and epithelial cells (podocytes), leading to proteinuria. The use of VEGF-R tyrosine kinase inhibitors can induce hand-foot syndrome, characterized by red spots, swelling, and pain on the extremities, particularly the palms or soles of the feet. This syndrome typically emerges within the first 6 weeks of treatment.

A meta-analysis has demonstrated that combination therapy with VEGF/VEGFR inhibitors yields superior survival benefits compared to chemotherapy for patients with PROC [42]. However, the trials included in the analysis encompassed recurrent OC rather than exclusively focusing on platinum-resistant disease, and they encompassed a subset of patients with platinum-sensitive disease as well. Moreover, the most recent clinical trials were not incorporated. Therefore, our study serves as a supplement to previous meta-analyses, offering more comprehensive content and considering more stratification factors. It also addresses the limitations of previous meta-analyses and provides additional treatment options for patients with PROC. Several limitations were encountered in this meta-analysis. Firstly, the RCTs employed various therapeutic agents and had different baseline characteristics, resulting in a high degree of heterogeneity in the data analysis for ORR. In an attempt to stratify based on baseline characteristics to mitigate heterogeneity, subgroup analyses were conducted. In the future, network meta-analysis can be employed to further investigate the efficacy and safety of combination therapy. Secondly, this study only included eight RCTs comparing VEGF/VEGFR inhibitors in combination therapy with chemotherapy in patients with PROC, and the majority of these trials were phase II trials. Further more reliable data would be provided from phase III clinical trials for analysis, especially when combined with VEGF/VEGFR inhibitors and PARP inhibitors, which are expected to be included in future studies. Additionally, it is important to note that this meta-analysis lacks sufficient subgroup analyses, and the inclusion of more stratification factors would be crucial in demonstrating the efficacy of VEGF/VEGFR inhibitors for PROC.

## Conclusions

The combination therapy of VEGF/VEGFR inhibitors for PROC has shown superior OS, PFS, and ORR compared to monochemotherapy, particularly when combined with VEGF/VEGFR inhibitors and chemotherapy. However, it is worth mentioning that combination therapy is associated with a higher incidence of certain adverse events, such as hypertension, mucositis, proteinuria, diarrhea, and hand-foot syndrome. Nevertheless, the safety profile of combination therapy remains manageable. The present study provides more treatment options for PROC patients.

### Electronic supplementary material

Below is the link to the electronic supplementary material.

Supplementary Material 1

## Data Availability

The original datasets for this study are included in the article/Supplementary Material.

## Abbreviations

OC: ovarian cancer
PROC: platinum-resistant ovarian cancer
PARP: poly (ADP-ribose) polymerase
PLD: pegylated liposomal doxorubicin
HR: hazard ratio
CI: confidence intervals
OS: overall survival
PFS: progression-free survival
OR: odds ratio
ORR: objective response rate
TRAEs: treatment-related adverse events
VEGF: vascular endothelial growth factor
ECOG: Eastern Cooperative Oncology Group
ASCO: American Society of Clinical Oncology
ESMO: European society of medical oncology
SGO: Society of Gynecologic Oncology
RCT: randomized controlled trial
RECIST 1.1: Response Evaluation Criteria in Solid Tumors version 1.1
GCIG CA125: Gynecological Cancer Intergroup cancer antigen 125
