# Supplementary material for: Efficacy and safety of VEGF/VEGFR inhibitors for platinum-resistant ovarian cancer: a systematic review and meta-analysis of randomized controlled trials
Source: BMC Womens Health. 2024 Jan 13;24:34. doi: 10.1186/s12905-023-02879-y (PMC10788010; doi:10.1186/s12905-023-02879-y)
Supplement: Supplementary file 1 — Supplementary Material 1 [file 12905_2023_2879_MOESM1_ESM.docx]

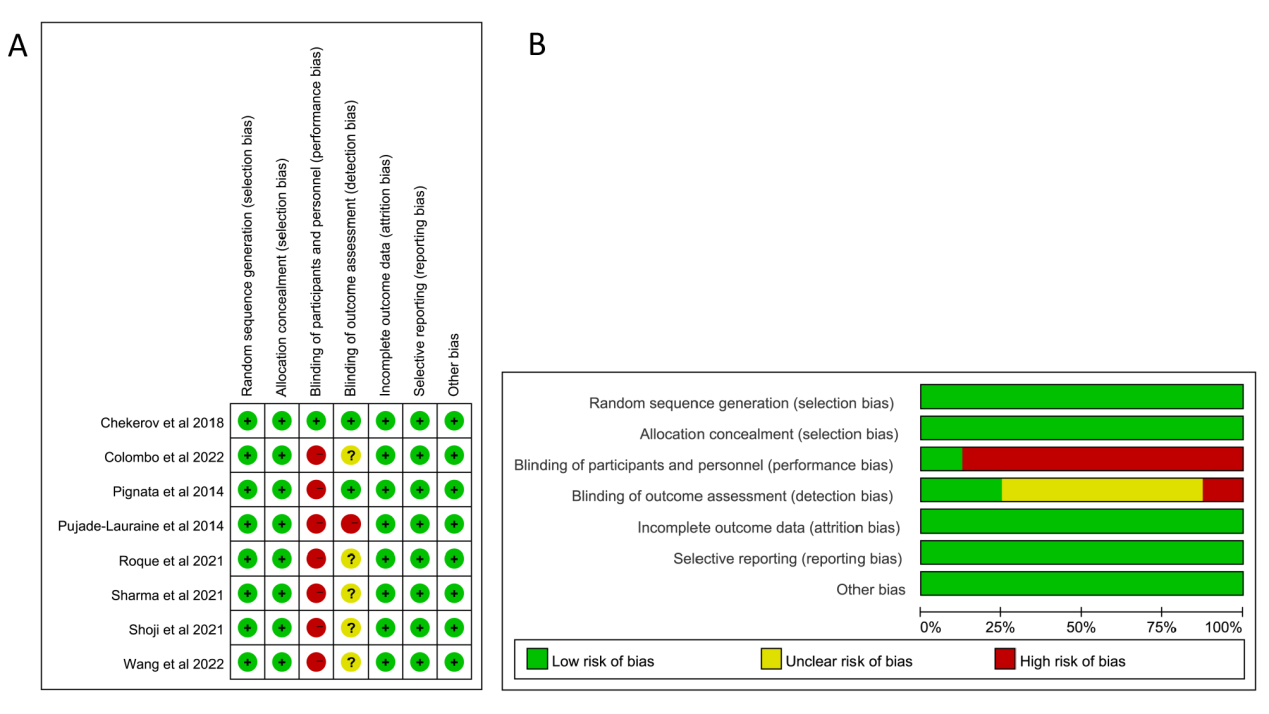


**Figure S1** Risk of bias summary (A) and risk of bias graph (B).


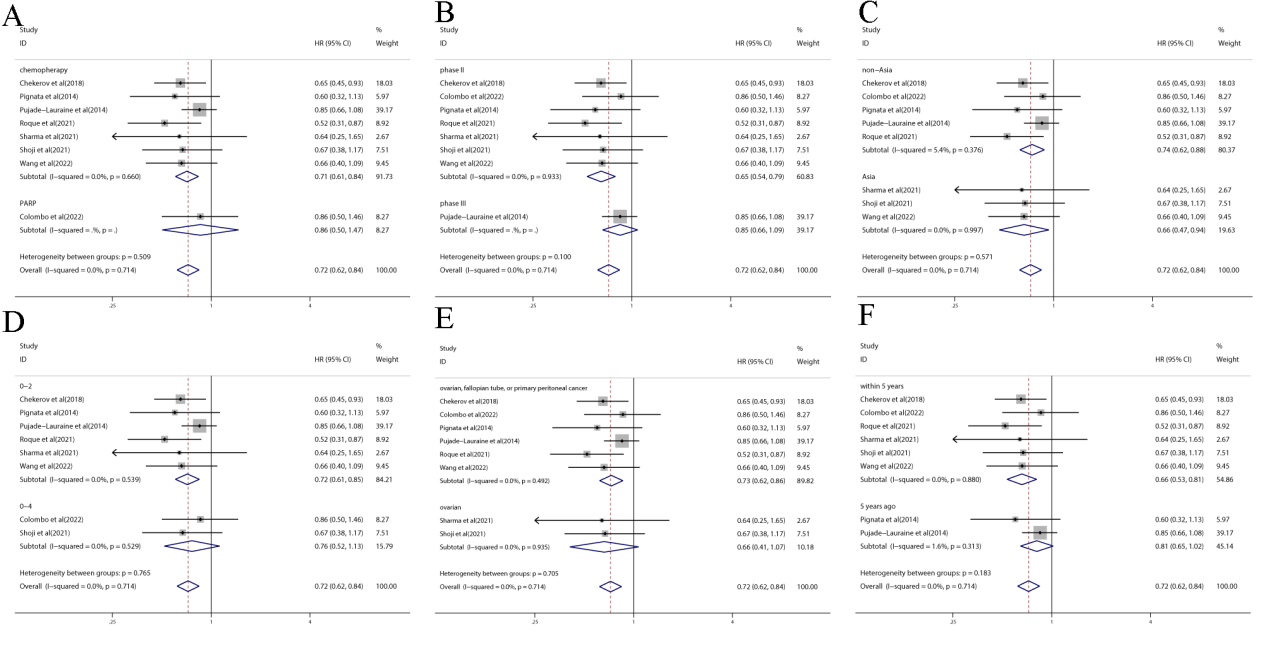


**Figure S2** Forest plots of OS of combination therapy with VEGF/VEGFR inhibitors in PROC in subgroup analysis (A) combination therapeutic agents, (B) trial phase, (C) region, (D) ECOG performance status, (E) primary tumor site, (F) publication year.


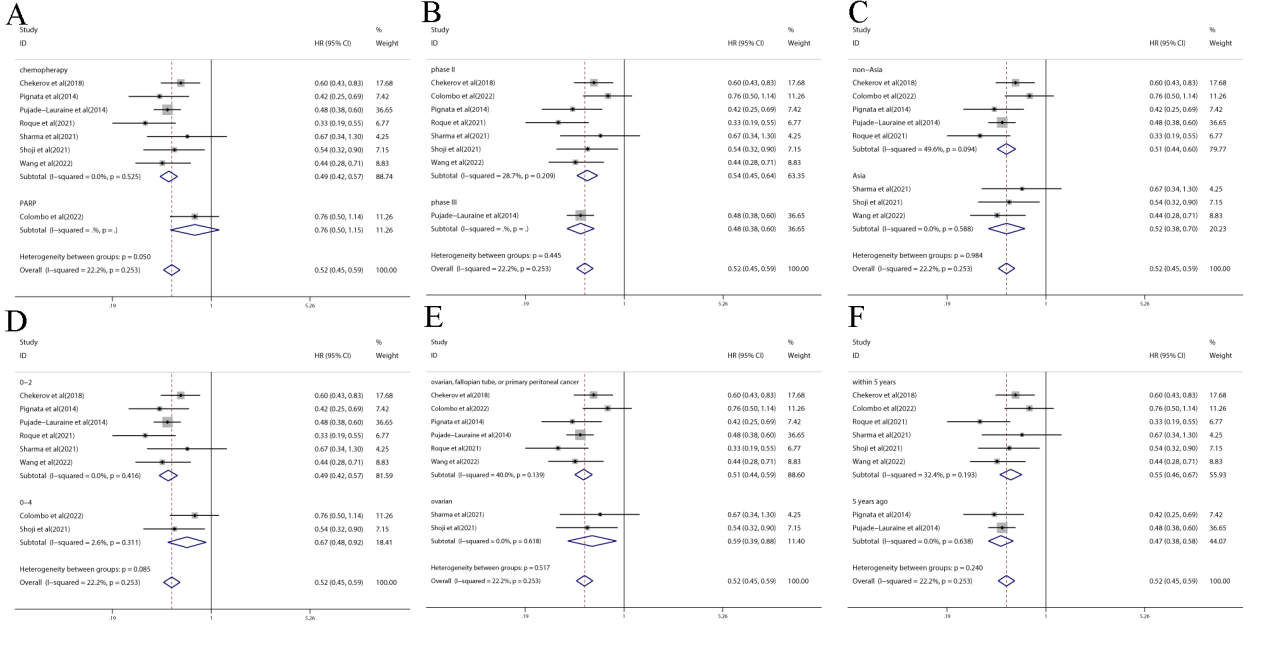


**Figure S3** Forest plot of PFS of combination therapy with VEGF/VEGFR inhibitors in PROC in subgroup analysis (A) combination therapeutic agents, (B) trial phase, (C) region, (D) ECOG performance status, (E) primary tumor site, (F) publication year.


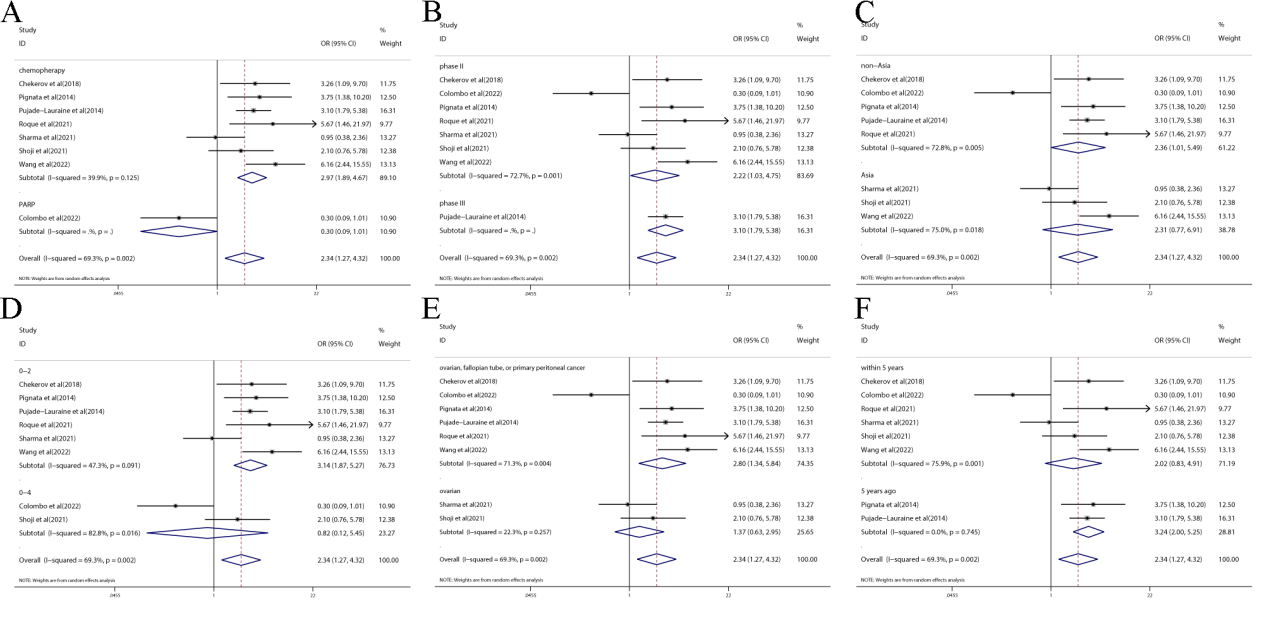


**Figure S4** Forest plot of ORR of combination therapy with VEGF/VEGFR inhibitors in PROC in subgroup analysis (A) combination therapeutic agents, (B) trial phase, (C) region, (D) ECOG performance status, (E) primary tumor site, (F) publication year.


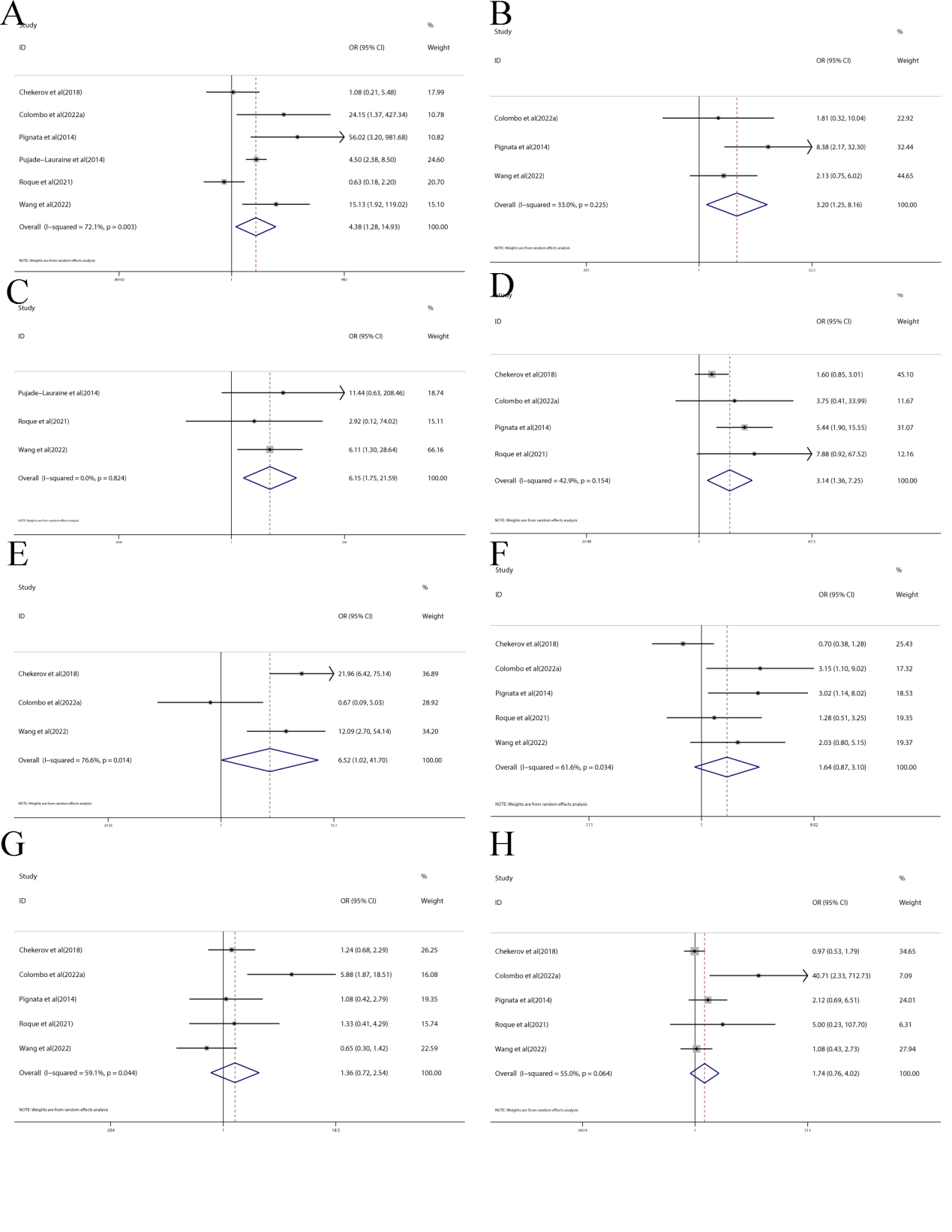


**Figure S5** Forest plot of TRAEs of combination therapy with VEGF/VEGFR inhibitors in PROC (A) Hypertension, (B) Mucositis, (C) Proteinuria, (D) Diarrhea, (E) Hand-foot syndrome, (F) Fatigue, (G) Nausea, (H) Vomiting.


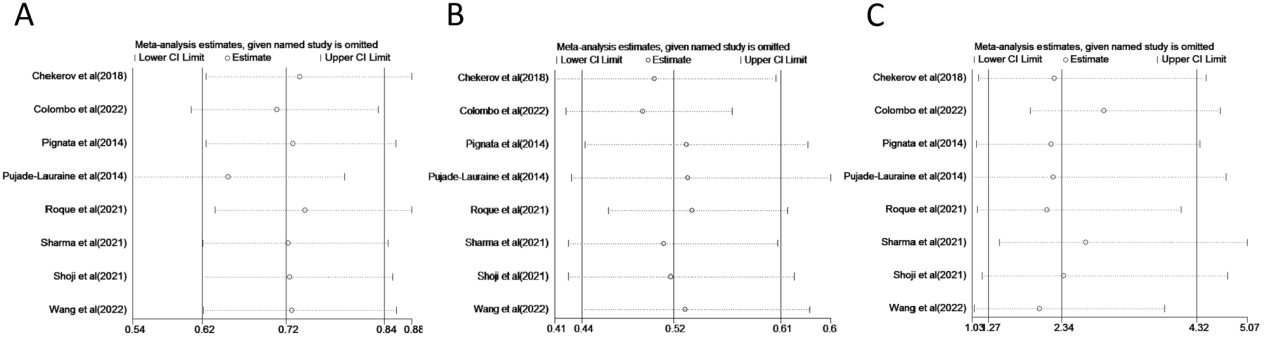


**Figure S6** Pooled HRs of OS (A), PFS (B) and ORR (C) in sensitivity analysis.


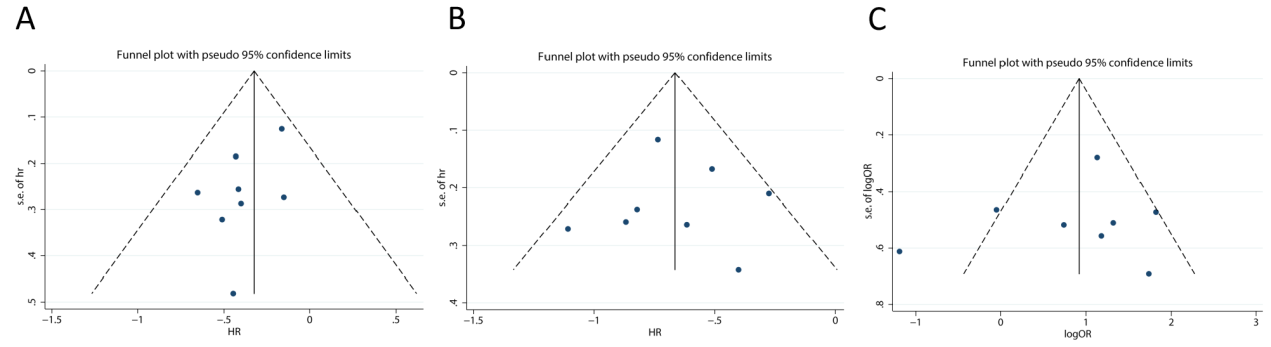


**Figure S7** Funnel plots for OS (A), PFS (B) and ORR (C).
